# An integrated approach to prioritizing areas for imported malaria re-establishment prevention in Chongqing, China

**DOI:** 10.3389/fcimb.2026.1929309

**Published:** 2026-10-19

**Authors:** Dan Jiang, Yan Tan, Mengping Yang, Shenbo Chen, Heyi Guan, Senping Yang, Huajun Bai, Fei Luo, Jun-Hu Chen, Shuang Zhou

**Affiliations:** 1 Chongqing Center for Disease Control and Prevention, Chongqing, China; 2 Liangjiang New Area Center for Disease Control and Prevention, Chongqing, China; 3 National Key Laboratory of Intelligent Tracking and Forecasting for Infectious Diseases, National Institute of Parasitic Diseases, Chinese Center for Diseases Control and Prevention (Chinese Center for Tropical Diseases Research); NHC Key Laboratory of Parasite and Vector Biology; WHO Collaborating Center for Tropical Diseases; National Center for International Research on Tropical Diseases, Shanghai, China

**Keywords:** imported malaria re-establishment, biomod2, China, Chongqing, spatial epidemiology

## Abstract

**Objective:**

This study integrated spatiotemporal analysis of imported malaria cases, ecological niche modeling of *Anopheles sinensis*, and field-measured vector density to prioritize county-level areas for imported malaria re‑establishment prevention in Chongqing, China. The findings facilitate a stratified and targeted response strategy, thereby providing evidence-based guidance for mitigating the risk of re-establishment arising from imported cases.

**Methods:**

Data on imported malaria cases in Chongqing (2014–2024) and vector surveillance records were compiled for this study. The vector data comprised two components: occurrence records (2011–2025) for ecological niche modeling and quantitative density measurements (2011–2024) for priority mapping, with density expressed as captures per trap-night during the peak mosquito activity season (June–October). Spatiotemporal pattern analysis was performed using R 4.5.2. Based on the vector data, ecological niche modeling was implemented via biomod2 (v.3.5.1), whereas all spatial operations—including map overlays and priority zoning—were conducted in ArcGIS 10.8.

**Results:**

Of the 301 imported malaria cases documented in Chongqing during 2014–2024, 75.08% were attributed to *P. falciparum* and 12.29% to *P. vivax*. The affected population was predominantly male (94.35%), aged 21–50 years (84.71%), and predominantly composed of outbound migrant workers (60.80%). Cases originated from 32 African countries (90.37%), with the Democratic Republic of the Congo and Nigeria accounting for the highest proportions. Spatially, cases were mainly clustered in the metropolitan area and western districts. Among the 39 *P. vivax* cases (including mixed infections), relative concentrations were observed in Yubei, Hechuan, and Pengshui, and 20 (51.28%) were reported during the peak mosquito activity season (June–October). The ensemble niche model for *An. sinensis* identified bio5, NDVI and bio11 as the leading environmental determinants, with predicted suitable habitats largely concentrated in the metropolitan and western areas. Field surveillance revealed substantial spatial heterogeneity in vector density, with the highest densities recorded in Wanzhou, followed by Yunyang, Fengjie, and Fengdu, whereas densities in the metropolitan districts were uniformly low except in Jiulongpo.

**Conclusions:**

Suitable habitats for *An. sinensis* were predicted across most of Chongqing, yet imported cases were primarily concentrated in the metropolitan area and western Chongqing. After further overlaying imported *P. vivax* cases and empirical vector density data. Wanzhou, Yubei, Hechuan, Tongnan, Pengshui, and Jiulongpo were identified as top-priority districts, whereas Tongliang, Shapingba, Jiangjin, Yongchuan, Dazu, Rongchang, Fuling, Wulong, Xiushan, Zhongxian, and Wushan were classified as moderate-priority districts. Enhanced surveillance should be prioritized to ensure timely detection and standardized clinical management of imported cases, coupled with vector suppression during the peak mosquito activity season—so as to eliminate opportunity for re-establishment.

## 1 Introduction

Following its malaria-free certification by the World Health Organization in 2021, China has since maintained zero indigenous transmission (Burki, 2021; Cao et al., 2021). However, the ongoing influx of imported cases from endemic regions, coupled with the widespread distribution of competent *Anopheles* vectors nationwide, constitutes a persistent threat of re‑establishment (Feng et al., 2022; Zhang et al., 2023; Yin, 2024). The convergence of imported infections, suitable vector habitats, and population mobility further amplifies this threat.

Chongqing is a mountainous municipality in southwestern China and features a humid subtropical climate. Historically, it was among the regions of China most affected by malaria, and all districts and counties reported cases, predominantly of *P. vivax*. From the 1950s onward, four major epidemic peaks occurred before transmission was largely brought under control in the 1990s. In 2010, Chongqing launched its elimination action plan, and no locally acquired cases have been reported since 2011. In 2019, it passed the national elimination assessment. Despite this achievement, Chongqing as a key node city along the “Belt and Road” Initiative has continuously expanded cooperation and exchanges with African countries and other malaria-endemic regions. This has resulted in a persistently high risk of imported malaria cases, mostly affecting returning travelers and outbound migrant workers who had traveled to sub-Saharan Africa and Southeast Asia (Fei et al., 2017; Fei et al., 2021; Wang et al., 2023).

Although no indigenous transmission has been recorded since elimination, the persistent inflow of imported cases continues to prompt concerns over re‑establishment. Among the historically recorded anopheline species in Chongqing, *An. sinensis* has been consistently identified as the predominant species across all surveyed districts based on sustained vector surveillance from 2011 to 2025, whereas no occurrence of *An. minimus* or *An. lesteri* has been documented during the same period. This predominance warrants particular attention. It is attributed, in part, to its broad habitat suitability, as the species thrives in diverse breeding sites (Ren et al., 2016), including rice paddies, irrigation ditches, streams, and marshes that are extensively distributed across Chongqing’s landscape. Its widespread resistance to pyrethroid insecticides, the cornerstone of global malaria vector control; this resistance substantially compromises the efficacy of standard interventions (Zhang et al., 2017; Zhao and Xue, 2024). As a primary vector of *P.vivax, An. sinensis* has established its key vector status in the post-elimination era through its ecological predominance, broad habitat suitability, and insecticide resistance. The sustained vector presence, coupled with frequent case importations, poses a non-negligible threat of re-establishment particularly in areas where environmental conditions are conducive to vector survival. Therefore, habitat suitability mapping for dominant *Anopheles* vectors is considered critical for assessing transmission potential under both current and future climatic regimes (Zayed et al., 2023; Yan et al., 2025). Species distribution models offer a practical means to this end; the ensemble approach implemented in the biomod2 package of R software (Li et al., 2024; Xu et al., 2025), for instance, integrates occurrence records with bioclimatic and topographic predictors to generate spatial predictions.

In this study, we integrated case surveillance (2014–2024), ecological niche modeling of *An. sinensis* (2011–2025), and field vector density (2011–2024) to prioritize county‑level areas for imported malaria re‑establishment prevention in Chongqing. The spatial overlap of these three lines of evidence identified priority districts to guide stratified and targeted surveillance in the post‑elimination context.

## 2 Materials and methods

### 2.1 Data collection

Imported malaria case data in Chongqing from January 1, 2014 to December 31, 2024 were obtained from the Infectious Disease Information Reporting Management System and the Parasitic Disease Information Reporting Management System. Vector surveillance data from 2011 to 2025 were collected through field monitoring across counties and districts of Chongqing. Field collections were conducted annually during the peak mosquito activity season (June to October) using light traps. These data included both occurrence records for niche modeling and quantitative density measurements (captures per trap-night). The occurrence records used for species distribution modeling are provided in **Supplementary Table 1**. The quantitative density data were aggregated at the district/county level and averaged over the peak seasons from 2011 to 2024 to align temporally with the case data. Key environmental factors potentially influencing the distribution of *Anopheles* mosquitoes were obtained from two sources. Historical climate data (1970–2000) were downloaded from WorldClim version 2.1 at a spatial resolution of 30 arc seconds (https://worldclim.org) (Wang et al., 2025; Yu et al., 2025). Enhanced Vegetation Index (EVI) and Normalized Difference Vegetation Index (NDVI) were acquired from the MODIS/061/MOD13A1 product via the NASA website (https://lpdaac.usgs.gov) (Guo et al., 2026), the full list of candidate environmental variables considered for modeling is provided (**Table 1**). The administrative base map of Chongqing was sourced from the National Center for Basic Geographic Information (https://www.tianditu.gov.cn/).

**Table 1:** Variables included in the model.

Category	Environmental Variable	Variable name	Unit	Participating in Modeling		
Bioclimatic	Annual mean air temperature	Bio1	°C			
	Mean diurnal range (Mean of monthly (max temp¨Cmin temp))	Bio2	°C	✓		
	Isothermality (Bio2/Bio7) (¡Á100)	Bio3	/			
	Variation in temperature seasonality	Bio4	°C			
	Max temperature of warmest month	Bio5	°C	✓		
	Min temperature of coldest month	Bio6	°C			
	Temperature annual range (BIO5¨CBIO6)	Bio7	°C			
	Mean temperature of wettest quarter	Bio8	°C			
	Mean temperature of driest quarter	Bio9	°C			
	Mean temperature of warmest quarter	Bio10	°C			
	Mean temperature of coldest quarter	Bio11	°C	✓		
	Annual precipitation	Bio12	mm	✓		
	Precipitation of wettest month	Bio13	mm			
	Precipitation of driest month	Bio14	mm	✓		
	Precipitation seasonality (Coefficient of variation)	Bio15	/			
	Precipitation of wettest quarter	Bio16	mm	✓		
	Precipitation of driest quarter	Bio17	mm	✓		
	Precipitation of warmest quarter	Bio18	mm	✓		
	Precipitation of coldest quarter	Bio19	mm			
Topographic	Elevation	Alt	M			
Vegetation	Annual normalized difference vegetation index	NDVI	/	✓		
	Enhanced Vegetation Index	EVI	/			

### 2.2 Statistical analysis

All statistical analysis were performed using R 4.5.2.

#### 2.2.1 Spatial thinning

To mitigate the risk of model overfitting arising from spatial autocorrelation among occurrence points, spatial thinning was performed on the vector occurrence data using the SDMToolbox v.2.5 extension within ArcGIS. Specifically, no more than one occurrence point was kept per 1 km × 1 km grid cell. This process yielded 127 valid occurrence points for use in the ensuing modeling steps (**Figure 1**).

**Figure 1:**
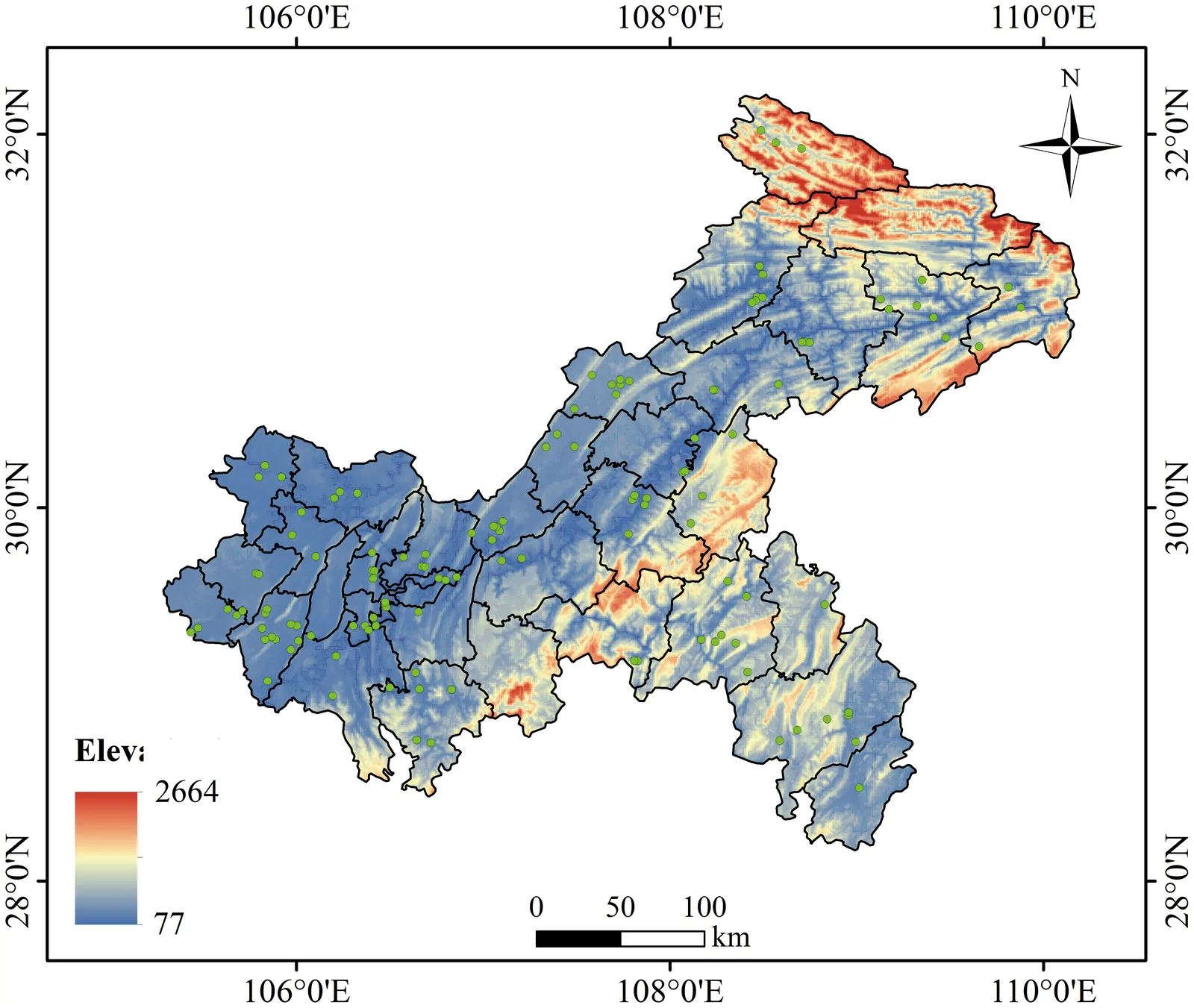
Spatially thinned occurrence points of *An. sinensis* in Chongqing.

#### 2.2.2 Environmental variable selection

To screen environmental variables prior to modeling, Pearson correlation analysis was conducted using the cor function in R. Variables with an absolute correlation coefficient |r|> 0.8 that were also of relatively low ecological importance were excluded from further consideration. 

#### 2.2.3 Ensemble modeling with biomod2

Ensemble ecological niche modeling was conducted using the biomod2 package (v.3.5.1), incorporating twelve algorithms: GLM, GBM, GAM, CTA, ANN, FDA, SRE, MARS, RF, XGBOOST, MAXNET and MAXENT. Default parameters were used for each algorithm. To ensure robustness, the occurrence data were randomly split into training (80%) and testing (20%) sets. A total of 254 pseudo-absence points were randomly generated, and this process was carried out five times. Each model was run 10 times, and a total of 562 individual models were successfully fitted after calibration (some algorithms were excluded under certain pseudo‑absence repetitions due to convergence issues). Model performance was evaluated using two metrics: the area under the receiver operating characteristic curve (AUC-ROC) and the true skill statistic (TSS) (Chen et al., 2026; Li et al., 2026). Only models with an ROC > 0.8 were retained for ensemble construction.

#### 2.2.4 Habitat suitability classification

The ensemble model output a continuous suitability index (*P*) ranging from 0 to 1000. We reclassified this index in ArcGIS using the Reclassify tool into four tiers: *P* < 200 as unsuitable, 200 ≤ *P* < 400 as low suitability, 400 ≤ *P* < 600 as moderate suitability, and P ≥ 600 as high suitability.

## 3 Results

### 3.1 Epidemiological profile

During the study period, 301 imported malaria cases were reported in Chongqing, with annual case numbers fluctuating between 4 and 48. The peak occurred in 2024 with 48 cases, representing 15.95% of the total. Only 22 cases were documented during the COVID‑19 pandemic (2020–2022); thereafter, an upward was observed (**Figure 2**). In terms of species distribution, *P. falciparum* was the predominant species, accounting for 226 cases (75.08%), followed by *P. vivax* (37, 12.29%), *P. ovale* (24, 7.97%), *P. malariae* (4, 1.33%), and mixed infections (10, 3.32%).

**Figure 2:**
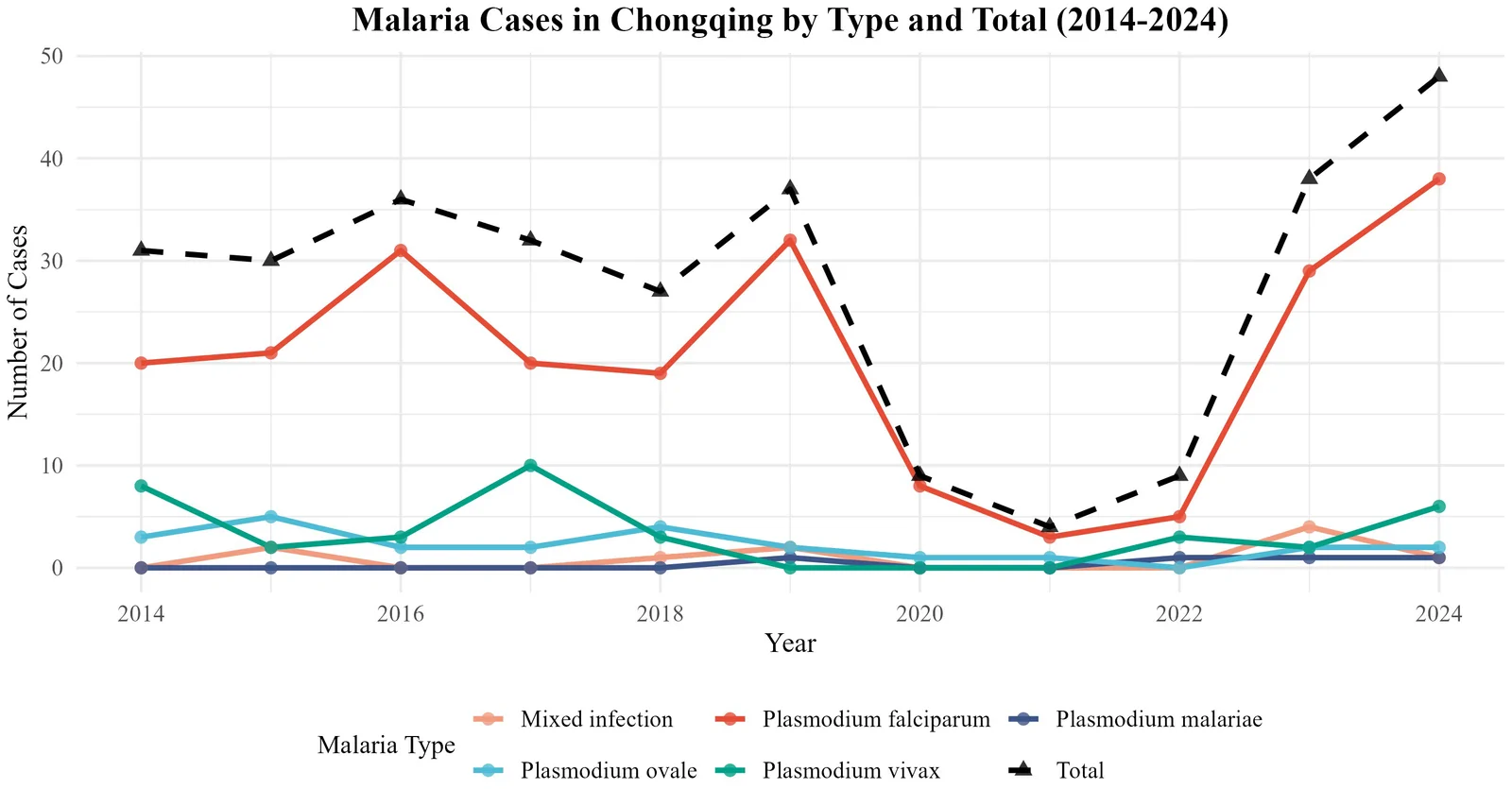
Imported malaria in Chongqing, 2014–2024.

The reported cases were predominantly in the 21–50 years age group, accounting for 84.71% (255/301), with a mean age of 38.75 ± 10.61 years. Males constituted 94.35% (284/301) of the cases. In terms of occupation, the majority of cases (60.80%, 183/301) were overseas laborers, including workers, migrant workers, and farmers (**Table 2**). Geographically, the imported cases originated from 32 African countries (272 cases, 90.37%), 4 Southeast Asian countries (16 cases, 5.32%), and other regions (13 cases, 4.32%). Among cases contracted in Africa, the highest numbers were recorded in the Democratic Republic of the Congo and Nigeria (44 cases each, each accounting for 16.18% of African cases), followed by Guinea (29 cases, 10.66%) (**Supplementary Figure 1**). Severe malaria was reported in 4 patients (1.33%), with three deaths.

**Table 2:** Demographic characteristics of imported malaria cases in Chongqing (N = 301).

Year	2014	2015	2016	2017	2018	2019	2020	2021	2022	2023	2024
Career											
Cadres/Staff	0	1	2	3	2	2	0	0	0	4	4
Commercial services	5	0	1	0	2	3	0	2	1	2	2
Farmers	3	13	6	3	5	9	2	0	1	9	6
Migrant workers	8	3	5	11	0	3	1	0	0	2	2
Others	5	6	8	4	8	9	1	1	4	9	19
Students	2	0	1	0	1	1	0	0	0	0	3
Workers	8	7	13	11	9	10	5	1	3	12	12
Age											
≤20	0	0	1	1	0	0	0	0	0	1	1
21-50	27	29	32	28	24	32	9	3	7	29	35
≥51	4	1	3	3	3	5	0	1	2	8	12
Gender											
Female	2	1	2	3	2	2	0	0	0	3	2
Male	29	29	34	29	25	35	9	4	9	35	46
Medical hospital level											
the International Travel Health Care Center	0	0	0	3	1	0	0	0	0	0	1
city-level hospitals	24	15	25	14	18	23	6	4	3	15	22
county-level hospitals	7	15	11	15	8	14	3	0	6	23	25
Time from onset to diagnosis											
>7 days	12	12	17	12	12	17	4	0	0	17	21
≤3 days	10	10	7	14	7	7	2	0	7	9	15
4–7 days	9	8	12	6	8	13	3	4	2	12	12
Total	31	30	36	32	27	37	9	4	9	38	48

### 3.2 Health-seeking behavior

The median time from illness onset to diagnosis was 5.6 days (IQR: 2.7–11.5 days) among all cases. Notably, 41.20% of cases were diagnosed more than seven days post‑onset. Regarding healthcare facility utilization, a total of 169 patients (56.15%) sought medical care in city‑level hospitals, and 127 (42.19%) in county‑level hospitals, and the remaining 5 patients received medical services in other healthcare facilities, such as international travel medical centers (**Table 2**).

### 3.3 Case distribution

Of the 301 imported malaria cases reported in Chongqing, 274 remained in Chongqing, whereas the other 27 traveled onward. Spatial analysis focusing on the 274 locally destined cases from 2014 to 2024 revealed cases were more frequently reported in the metropolitan area and the western districts of Chongqing. Cases were documented across all counties and districts except Shizhu County. Six districts, specifically Tongnan, Hechuan, Yubei, Shapingba, Yongchuan, and Nan’an, each reported more than 10 cases (**Supplementary Figure 2**).

Given that *An. sinensis* is the primary vector for *Plasmodium vivax* malaria in the region, we further examined imported *P. vivax* cases to assess vector-relevant transmission risk. Among the 39 imported *P. vivax* cases (including *P. vivax* mixed infections) identified during the same period, spatial mapping by district/county of residence revealed that cases were predominantly concentrated in Yubei, Hechuan, and Pengshui, followed by Bishan, Tongnan and Jiulongpo (**Figure 3**). Notably, 20 of these 39 cases (51.28%) were reported during the peak mosquito activity season (June–October).

**Figure 3:**
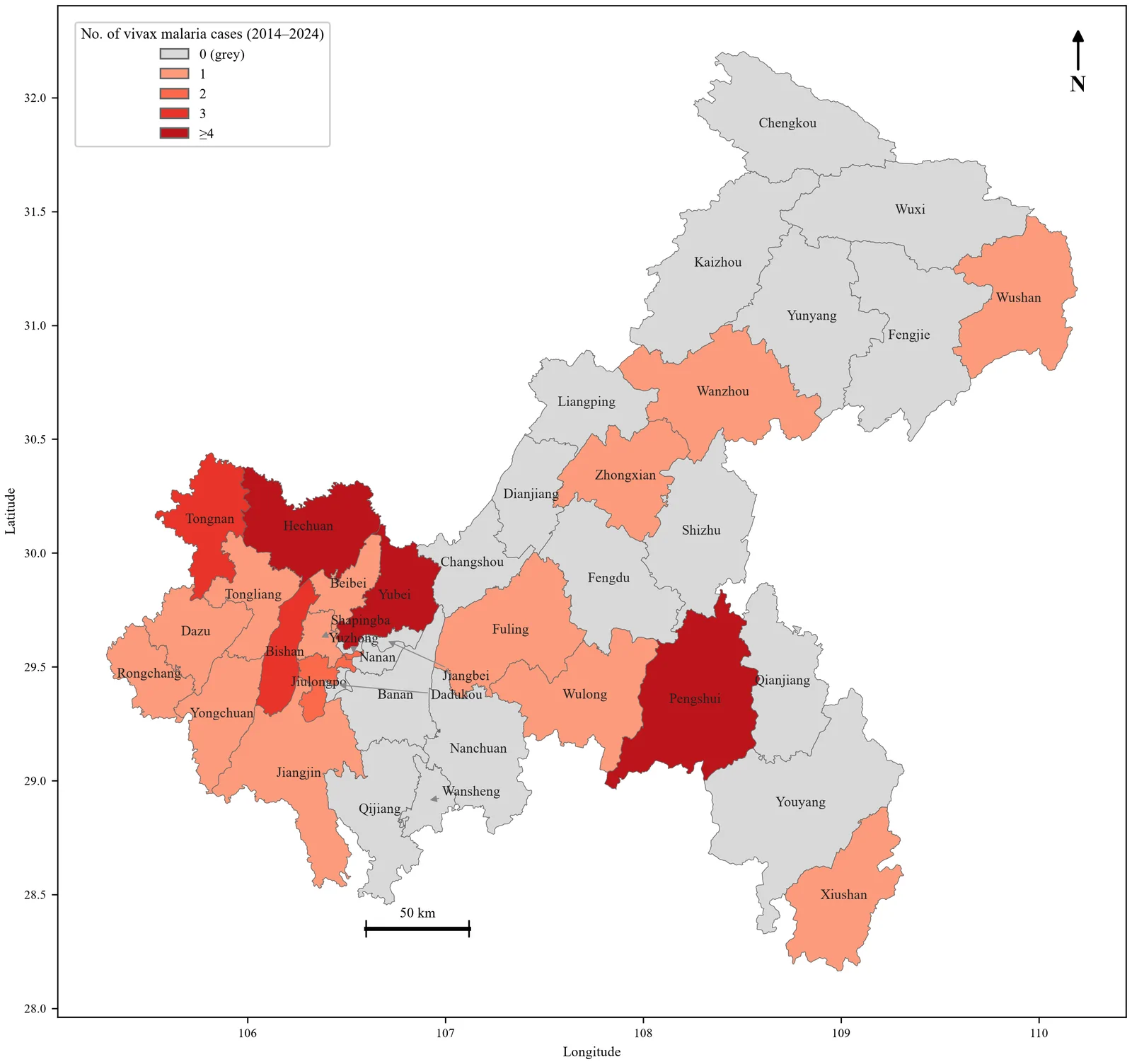
Spatial distribution of imported *P. vivax* cases in Chongqing, 2014–2024.

### 3.4 Potential distribution of *An. sinensis* and analysis of model environmental variables

#### 3.4.1 The variables for modeling

Pearson correlation analysis was conducted to screen the candidate environmental variables. Variables with an absolute correlation coefficient |r|> 0.8 that also exhibited relatively low ecological importance were excluded from the final model. Ultimately, nine variables were retained: bio2, bio5, bio11, bio12, bio14, bio16, bio17, bio18, and NDVI (**Figure 4**, **Table 1**).

**Figure 4:**
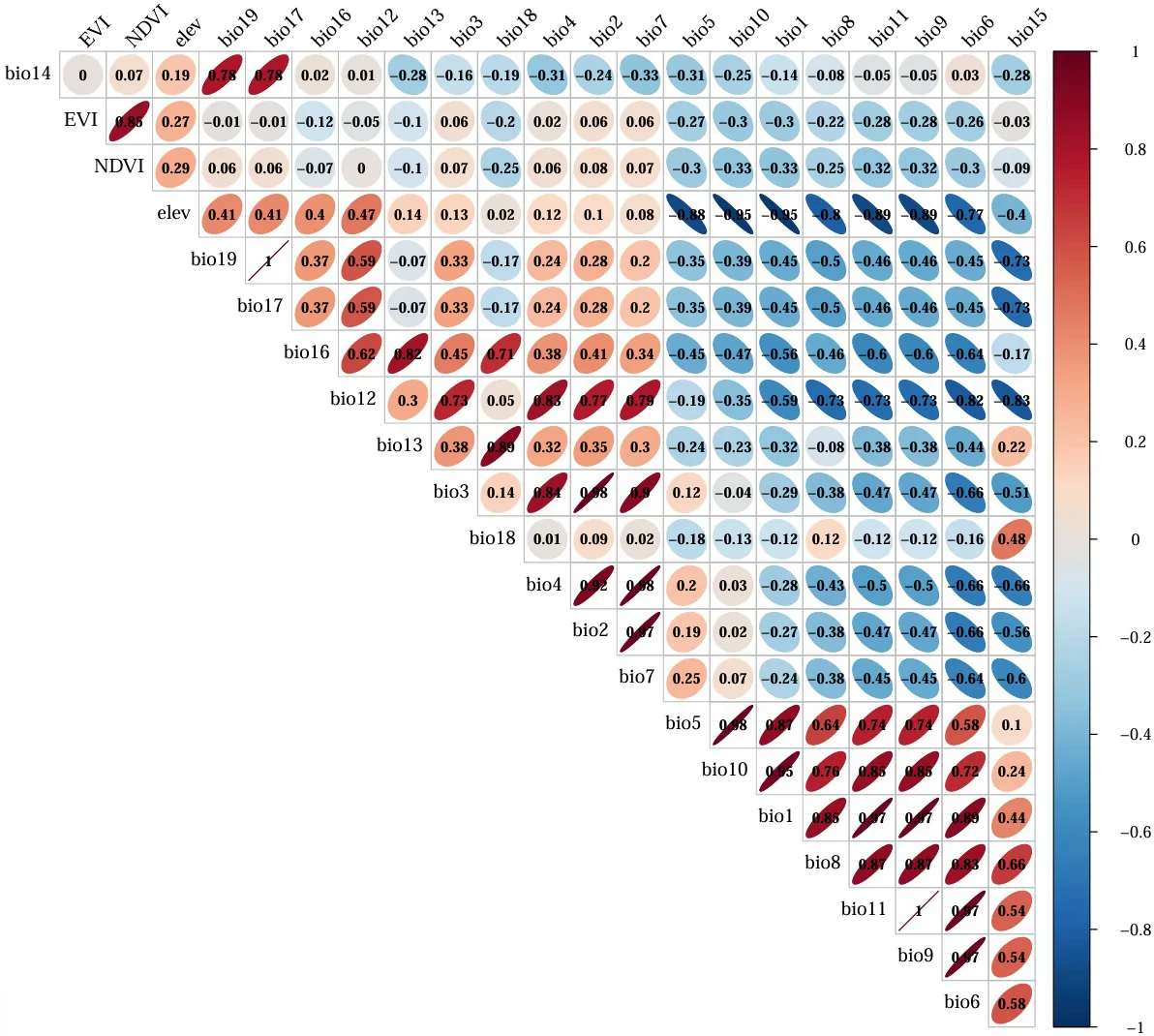
Pearson correlation analysis of variables.

#### 3.4.2 Model performance

This study used ROC and TSS values to evaluate the performance of individual and ensemble models (**Figure 5**). Among the 12 individual models, RF achieved the highest performance (mean ROC = 1, mean TSS = 1), followed by XGBOOST (mean ROC = 0.9517, mean TSS = 0.8218) and GBM (mean ROC = 0.8256, mean TSS = 0.5095) (**Table 3**, **Figure 5**). The ensemble model built via Biomod2 (ROC = 0.839, TSS = 0.531) outperformed the individual models, indicating a substantial improvement in predictive performance.

**Figure 5:**
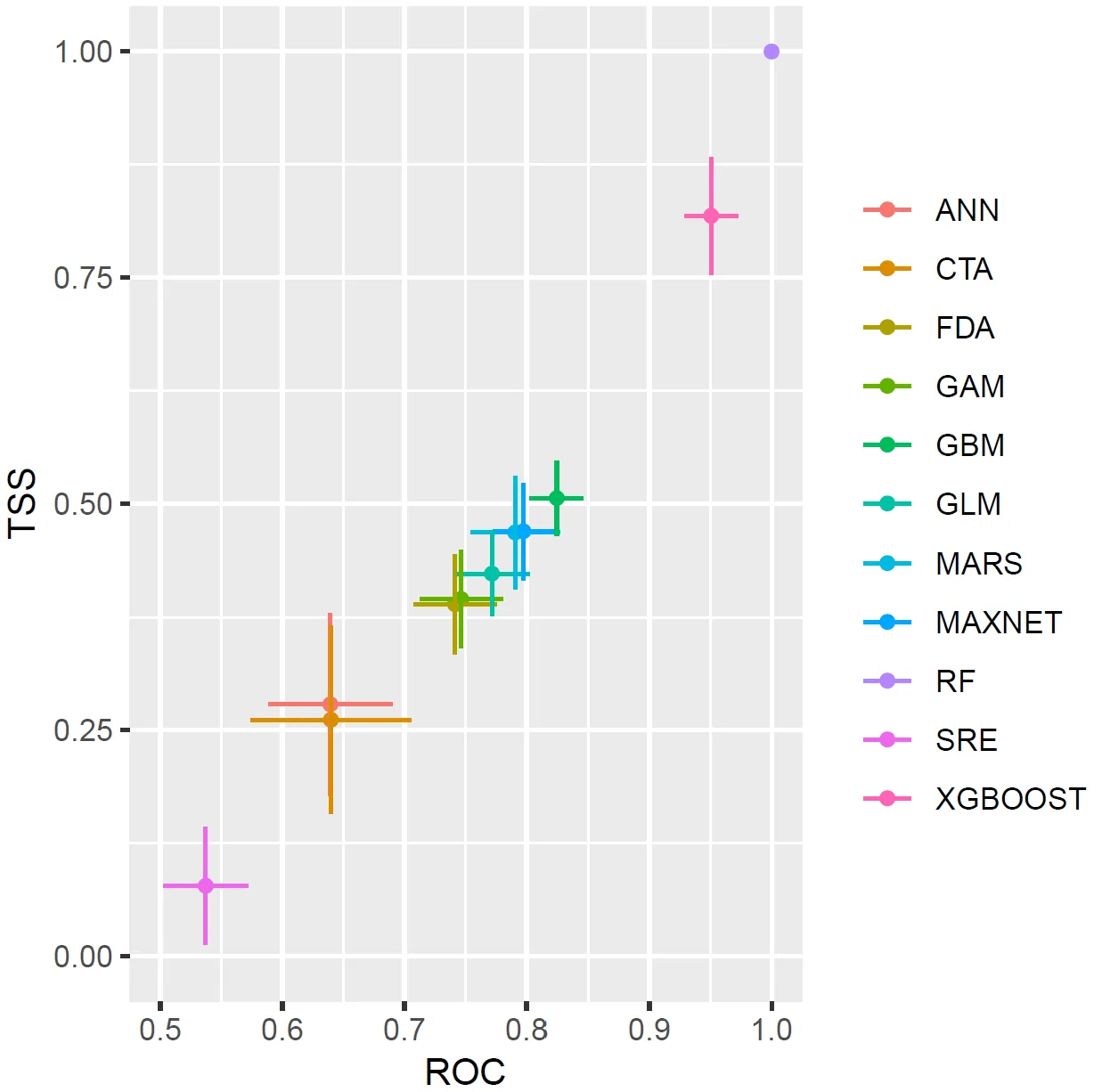
Values of ROC and TSS for a single model.

**Table 3:** Mean values of ROC and TSS for a single model.

Model	TSSmean	ROCmean	TSSsd	ROCsd
ANN	0.235	0.6175	0.0948	0.0474
CTA	0.2661	0.6416	0.1076	0.0671
FDA	0.3899	0.7412	0.0567	0.0346
GAM	0.3957	0.7465	0.0557	0.0348
GBM	0.5095	0.8256	0.0407	0.0211
GLM	0.4249	0.7722	0.0473	0.0315
MARS	0.4683	0.7911	0.0635	0.0373
MAXENT	0.4702	0.7979	0.0531	0.0249
RF	1	1	0	0
SRE	0.0617	0.5289	0.0465	0.0262
XGBOOST	0.8218	0.9517	0.0636	0.0208

#### 3.4.3 Variable contribution

Nine environmental variables were incorporated into the model. Their normalized contribution weights were calculated within the biomod2 framework. Bio11, bio5, and NDVI showed the highest contributions, collectively accounting for most of the total weight (**Figure 6**).

**Figure 6:**
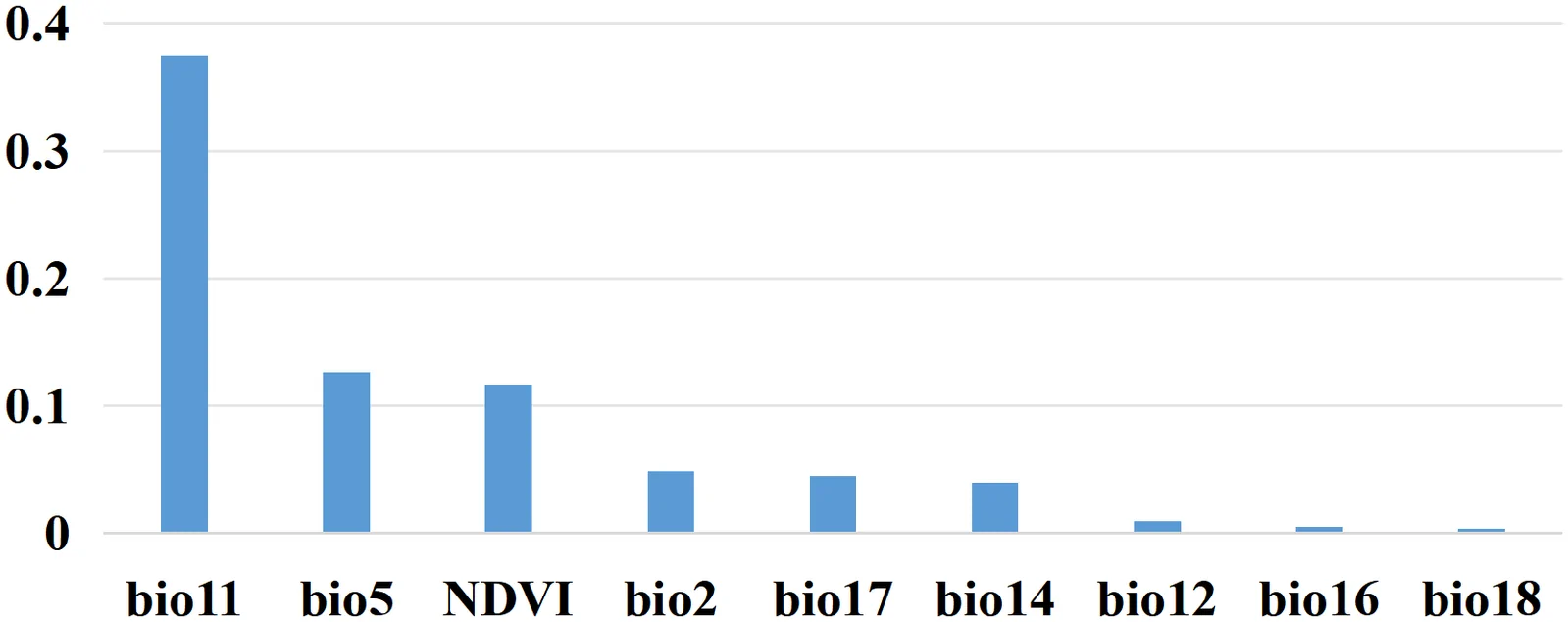
Relative contribution of environmental variables in ensemble model.

#### 3.4.5 The suitable distribution area of *An. sinensis*

Most areas of Chongqing were identified as suitable habitats, among which the highly suitable areas had the largest distribution. Unsuitable habitats were mainly concentrated in regions such as Chengkou, Wuxi, Shizhu, Fengdu, Wulong, and Nanchuan. In contrast, suitability levels in the southern, northern, and eastern parts of the municipality were relatively lower compared to western Chongqing (**Figure 7**).

**Figure 7:**
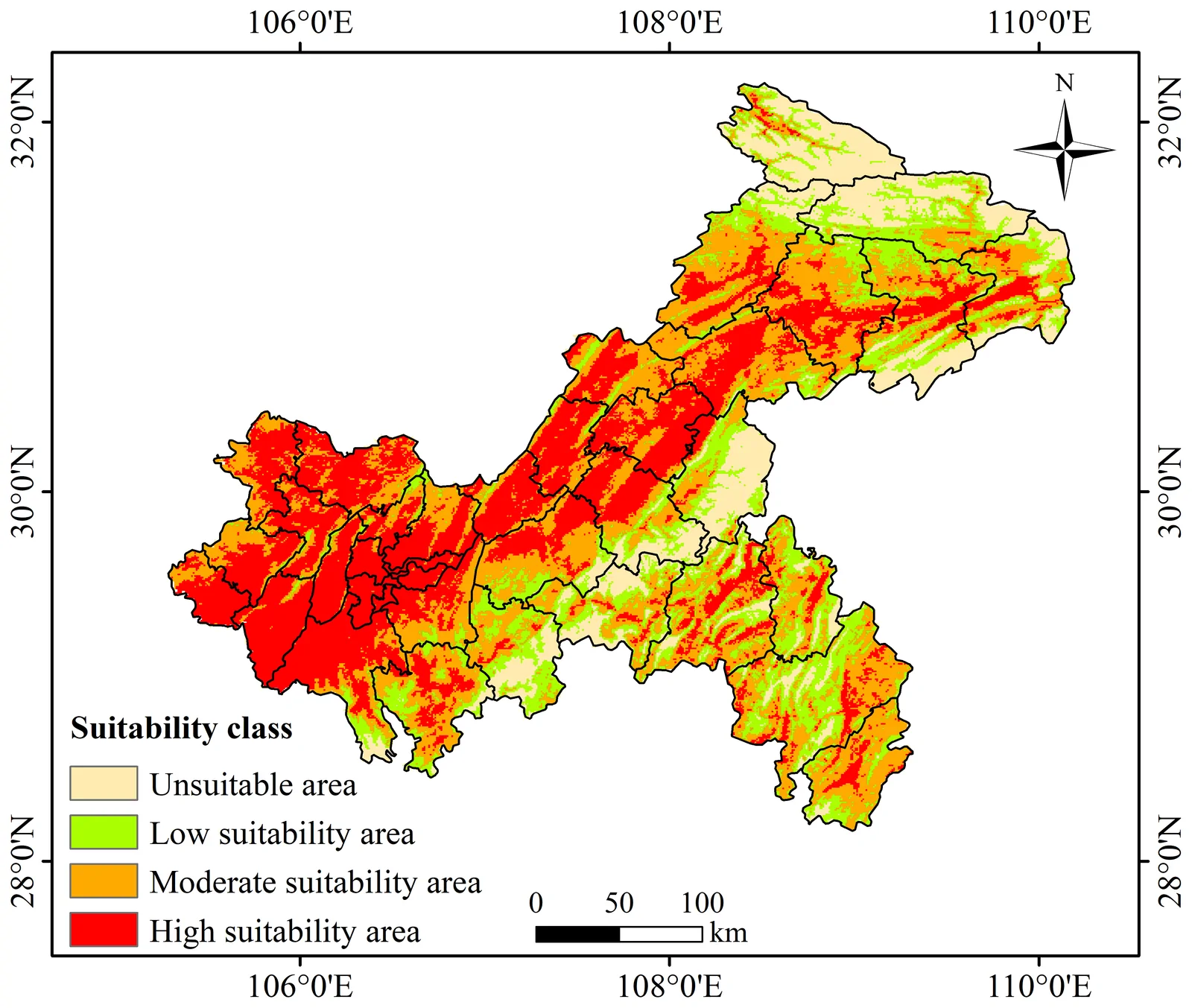
Fitting suitable distribution areas of *An.sinensis* in Chongqing.

#### 3.4.6 Spatial distribution of vector density

Although habitat suitability modeling identifies areas with favorable environmental conditions for vector presence, it does not directly reflect actual vector abundance, which is a more immediate determinant of transmission potential. To address this gap, the predicted suitability map was overlaid with field-measured densities of *An. sinensis* at the district/county level. As shown in **Figure 8**, the peak-season mean density of *An. sinensis* (captures per trap-night, 2011–2024) exhibited substantial spatial heterogeneity. Highest densities was observed in Wanzhou, followed by Yunyang, Fengjie and Fengdu. By contrast, densities in the metropolitan districts were uniformly low, except in Jiulongpo, where moderately higher densities were recorded. No *An. sinensis* were captured in light-trap surveys in Beibei, Bishan, Nan'an, Nanchuan, Wansheng and Wuxi.

**Figure 8:**
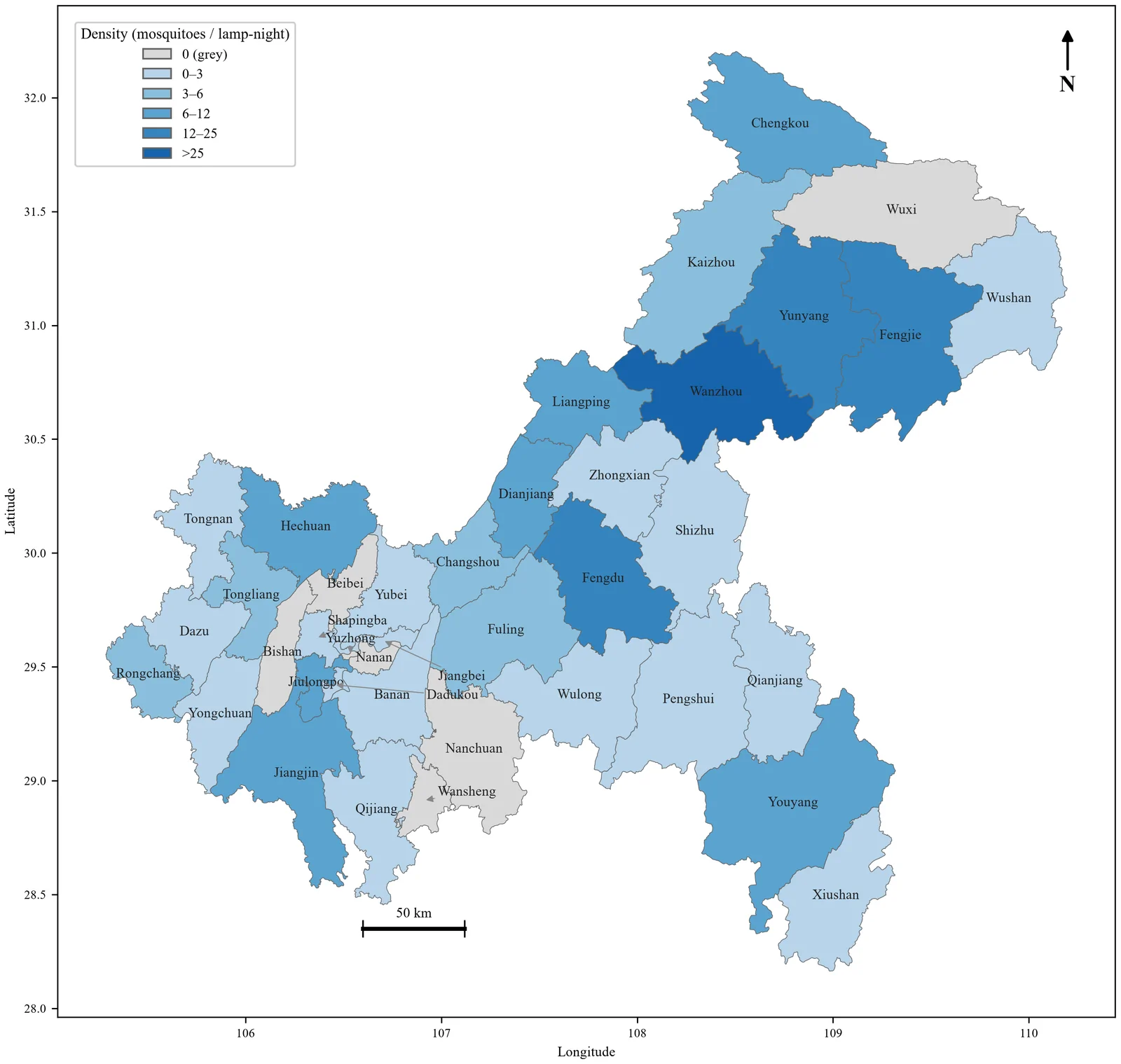
Density of *An. sinensis* (per trap-night) at the district/county level in Chongqing, 2011–2024.

## 4 Discussion

This study systematically characterized the epidemiological features of imported malaria in Chongqing from 2014 to 2024 and projected the potential distribution of the predominant *Anopheles* vectors through ecological niche modeling. This is the first study at the municipal level in Chongqing to spatially integrate imported case data with vector habitat suitability and field‑measured vector density, offering a scientific foundation for targeted interventions in the post-elimination era. In recent years, imported malaria cases in Chongqing have shown an upward trajectory, predominantly originating from sub‑Saharan Africa, with *P. falciparum* and *P. vivax* as the dominant species. This pattern may be associated with travel by outbound migrant workers from Chongqing under the Belt and Road Initiative, underscoring the pivotal role of population mobility in disease importation (Kang et al., 2022; Li et al., 2026). Movement restrictions during the early COVID‑19 pandemic led to a marked decline in cases, yet a rapid rebound followed, culminating in a peak of 48 cases in 2024. This pattern indicates that sustained surveillance among returning travelers remains essential, even amidst future global health emergencies (Lu et al., 2022; Liu et al., 2023). In terms of demographic profile, the affected population was predominantly male (94.35%) and aged 21–50 years (84.71%), with outbound migrant workers comprising 60.80% of all cases. This distribution aligns with findings from other imported malaria studies across China, including Yunnan and Anhui provinces (Yang et al., 2021; Liu et al., 2026), reflecting that young to middle‑aged males constitute the primary cohort engaged in overseas employment. Health education should therefore be prioritized for this demographic, covering prophylactic medication before departure and prompt medical consultation upon febrile illness after return (Wang et al., 2022; Obeagu et al., 2025). The median interval from symptom onset to diagnosis was 5.6 days, and 41.20% of patients were diagnosed more than seven days after symptom onset. Possible explanations for delayed diagnosis include low malaria awareness among patients, limited diagnostic capacity at county‑level hospitals, and the atypical or mild symptoms often associated with non‑falciparum malaria (e.g., *P. vivax* and *P. ovale*). Therefore, it is recommended that routine malaria screening be strengthened among febrile patients at county‑level hospitals to reduce diagnostic delays (Jia et al., 2024). Spatially, imported malaria cases in Chongqing were predominantly clustered in the metropolitan area and the western districts (**Figure 3** and **Supplementary Figure 2**). These regions are economically vibrant, densely populated, and serve as primary catchment areas for outbound migrant workers. Several locality‑specific factors further facilitate case importation: abundant medical resources in Shapingba, a large international student population in Nan’an, and well‑developed transport networks in Yubei collectively increase international travel and exposure risk.

*Anopheles* mosquitoes remain a major potential threat to malaria control and elimination (Hemingway, 2014; Ochomo et al., 2024; Obeagu et al., 2025). In recent decades, socioeconomic development has substantially reshaped living conditions and environmental landscapes, while ecological and climatic changes have concurrently altered the suitable distribution of vectors (Franklinos et al., 2019; Semenza et al., 2022). Using the biomod2 ensemble model, we predicted that highly suitable habitats for *An. sinensis* were primary located in the metropolitan area, western Chongqing, and surrounding districts. Among the environmental predictors, bio5, NDVI and bio11 had the largest relative contributions (Tong et al., 2023; Liu et al., 2026). Moderate NDVI values reflect suitable vegetation cover and adequate soil moisture, which offer favorable resting and oviposition sites. Conversely, excessively high NDVI, as seen in dense forests, may be less conducive to vector presence (Lippi et al., 2023). Bio11 captures the limiting effect of low winter temperatures on mosquito survival and overwintering. Higher winter temperatures in western Chongqing favor overwintering success and early spring resurgence of vector populations. In contrast, suitability was markedly lower in the eastern mountainous areas, such as Wuxi and Chengkou, where high altitude and low temperatures restrict vector distribution.

Marked spatial heterogeneity was observed in the distribution of *P. vivax* cases, with a relative concentration identified in Yubei, Hechuan, and Pengshui. This pattern is likely driven by the distinct connectivity profiles of these districts: Yubei serves as the primary entry point for international travelers via Jiangbei International Airport, whereas Hechuan and Pengshui, both traditional labor‑sending areas, may have higher proportions of migrant workers returning from *P. vivax*‑endemic regions, a factor contributing to their elevated importation risk. The fact that half of *P. vivax* cases arrive during the high-transmission season underscores the need for temporally targeted interventions, particularly enhanced surveillance and rapid response measures during the summer-autumn months. This geographic overlap between concentrated case importation and high vector suitability implies that transmission could re-establish if imported cases arrive during the vector-active season, provided that local vector abundance is sufficient to support transmission.

The predicted suitability broadly aligns with the known distribution of *An. sinensis*. Ecological niche models characterize climatic suitability—approximating the fundamental niche—rather than vector abundance, which is additionally shaped by local-scale factors such as breeding-site availability and land use. Consequently, the metropolitan area, despite its high climatic suitability, supports low vector density, as urbanization and sustained vector control limit available larval habitats, whereas Wanzhou, Yunyang, Fengjie, and Fengdu, among others, are major rice-growing areas whose extensive paddies sustain high *An. sinensis* abundance; Overlaying imported *P. vivax* cases with field‑measured vector density identified Wanzhou, Yubei, Hechuan, Tongnan, Pengshui, and Jiulongpo as priority districts. In addition, eleven districts—Tongliang, Shapingba, Jiangjin, Yongchuan, Dazu, Rongchang, Fuling, Wulong, Xiushan, Zhongxian, and Wushan—were classified as moderate-priority.

Several limitations of this study should be acknowledged. First, *An. minimus* and *An. lesteri* were not detected in our surveillance records and were therefore excluded from our assessment, although these species may persist at very low densities or in unsampled microhabitats; future investigations incorporating enhanced trapping and molecular methods could help clarify their status in Chongqing. Second, the limited number of imported *P. vivax* cases precluded robust quantitative risk estimation at the district/county level, the biological significance of case–vector overlap in the post‑elimination setting is not entirely contingent upon case frequency. Third, our overlay analysis was conducted at the district/county scale, which may mask finer‑scale heterogeneity at the township/village level, potentially leading to over‑ or underestimation of local risk. Fourth, field vector density data were collected during specific time periods and may not fully represent seasonal or interannual fluctuations. Finally, ensemble model predictions carry inherent uncertainty, particularly in areas with sparse occurrence records, which may affect habitat suitability assessments; nevertheless, the direct incorporation of empirical vector density data provided a complementary measure of observed vector abundance. Despite these limitations, our screening framework offers a practical, evidence‑based reference for identifying priority districts in Chongqing that require enhanced surveillance and targeted vector control.

Based on these findings, a tiered and targeted malaria surveillance system should be established for Chongqing. In districts where imported cases and vector density overlap at high levels, microscopy capacity at primary care facilities should be reinforced to enable earlier detection and faster response. For migrant workers returning to priority from malaria-endemic areas, pre‑departure health education and post‑arrival active screening are recommended. Additionally, seasonal vector density monitoring, should be implemented to support prevention‑oriented control.

## 5 Conclusion

Although no indigenous malaria cases have been documented in Chongqing since elimination, the threat of re‑establishment persists and exhibits marked regional heterogeneity. Imported cases were predominantly concentrated in the metropolitan area and western Chongqing—regions that also emerged as the most suitable habitats for *An.sinensis* in our ensemble model. However, after integrating the distribution of imported *P. vivax* cases and field‑measured vector density, a more refined priority pattern emerges. Wanzhou, Yubei, Hechuan, Tongnan, Pengshui, and Jiulongpo were identified as top-priority districts, where imported cases, vector suitability, and vector density overlap most pronouncedly, whereas Tongliang, Shapingba, Jiangjin, Yongchuan, Dazu, Rongchang, Fuling, Wulong, Xiushan, Zhongxian, and Wushan were classified as moderate-priority districts warranting continued surveillance. Enhanced surveillance and response capacity should be prioritized, with emphasis on timely case detection, active screening of returning migrant workers, and seasonal vector control during the peak mosquito activity season. Strengthening targeted interventions in high‑priority zones is essential to consolidate the gains of malaria elimination and avert potential re-establishment.

## Data Availability

The original contributions presented in the study are included in the article/**Supplementary Material**. Further inquiries can be directed to the corresponding authors.

## Supplementary material

Supplementary Table 1 The occurrence records used for species distribution modelling.

Supplementary Figure 1 Heatmap of imported malaria cases in Chongqing, China (2014–2024), by year and country of origin. Case counts are color-coded (light orange to dark red). Only countries with at least three cases in total are shown.

Supplementary Figure 2 Spatiotemporal distribution analysis of imported malaria cases in Chongqing.
